# Silent voices of the midwives: factors that influence midwives’ achievement of successful neonatal resuscitation in sub-Saharan Africa: a narrative inquiry

**DOI:** 10.1186/s12884-021-04339-7

**Published:** 2022-01-16

**Authors:** Jan Becker, Chase Becker, Florin Oprescu, Chiung-Jung (Jo) Wu, James Moir, Meshak Shimwela, Marion Gray

**Affiliations:** 1 Midwife Vision Global Ltd, PO BOX 9165, Pacific Paradise, QLD 4564 Australia; 2 University of the Sunshine Coast, 90 Sippy Downs Dr, Sippy Downs, QLD 4556 Australia; 3 General Division of the Order of Australia, Office of the Official Secretary to the Governor-General, Government House, ACT 2600 Canberra, Australia; 4 University of Nicosia Medical School in Partnership with St George’s University of London, Makedonitissis 46, Nicosia, 2417 Cyprus; 5 School of Health and Behavioural Sciences, University of Sunshine Coast (USC), 90 Sippy Downs Dr, Sippy Downs, QLD 4556 Australia; 6 School of Nursing, Midwifery and Paramedicine, University of the Sunshine Coast (USC), 1 Morton Bay Parade, Petrie, QLD 4502 Australia; 7 Royal Brisbane and Women’s Hospital, Butterfield St, Herston, QLD 4029 Australia; 8 Clinical Director, QLD Fertility Group Sunshine Coast, 44 Clarkes Road, Diddillibah, QLD 4559 Australia; 9 Internal Medicine, Temeke Regional Referral Hospital, Temeke Road, Adjacent Sterio Market, Dar es Salaam, Tanzania; 10 Centre for Health Research/School of Health and Wellbeing; Associate Dean (Clinical), Faculty of Health, Engineering and Sciences, University of the Southern Queensland, Sinnathamby Blvd, Springfield Central, Queensland 4300 Australia

**Keywords:** Very early neonatal death, Narrative inquiry, Sub-Saharan Africa, Self-efficacy, Clinical practice, Neonatal resuscitation, Stories, Simulation, Limited clinical resources, Midwives’ voices

## Abstract

**Background:**

In Tanzania, birth asphyxia is a leading cause of neonatal death. The aim of this study was to identify factors that influence successful neonatal resuscitation to inform clinical practice and reduce the incidence of very early neonatal death (death within 24 h of delivery).

**Methods:**

This was a qualitative narrative inquiry study utilizing the 32 consolidated criteria for reporting qualitative research (COREQ). Audio-recorded, semistructured, individual interviews with midwives were conducted. Thematic analysis was applied to identify themes.

**Results:**

Thematic analysis of the midwives’ responses revealed three factors that influence successful resuscitation:

1. Hands-on training (“HOT”) with clinical support during live emergency neonatal resuscitation events, which decreases fear and enables the transfer of clinical skills;

2. Unequivocal commitment to the Golden Minute® and the mindset of the midwife; and.

3. Strategies that reduce barriers.

Immediately after birth, live resuscitation can commence at the mother’s bedside, with actively guided clinical instruction. Confidence and mastery of resuscitation competencies are reinforced as the physiological changes in neonates are immediately visible with bag and mask ventilation.

The proclivity to perform suction initially delays ventilation, and suction is rarely clinically indicated. Keeping skilled midwives in labor wards is important and impacts clinical practice. The midwives interviewed articulated a mindset of unequivocal commitment to the baby for one Golden Minute®. Heavy workload, frequent staff rotation and lack of clean working equipment were other barriers identified that are worthy of future research.

**Conclusions:**

Training in resuscitation skills in a simulated environment alone is not enough to change clinical practice. Active guidance of “HOT” real-life emergency resuscitation events builds confidence, as the visible signs of successful resuscitation impact the midwife’s beliefs and behaviors. Furthermore, a focused commitment by midwives working together to reduce birth asphyxia-related deaths builds hope and collective self-efficacy.

**Supplementary Information:**

The online version contains supplementary material available at 10.1186/s12884-021-04339-7.

## Background

Approximately 2.4 million neonatal deaths occur worldwide each year [1]. Ninety-nine percent of these deaths occur in low- and middle-income countries [2]. Birth asphyxia-related neonatal deaths (now called intrapartum-related neonatal deaths) account for approximately 1 million deaths annually [3] and are defined by the World Health Organization (WHO) as death resulting from “failure to initiate and sustain breathing at birth” [4].

Worldwide, 36.3% of all neonatal deaths occur within the first 24 h after birth (within 24 h of delivery), and these are referred to as very early neonatal deaths (VENDs) [3]; most of these deaths occur in sub-Saharan African countries [5–7].

In Tanzania, birth asphyxia is a leading cause of death, with a rate of 40,000 neonates per annum [5]. Thirty percent of neonatal deaths related to intrapartum-related hypoxia (birth asphyxia) [6] are due to a lack of interventions at birth, such as stimulation, warmth, and bag and mask ventilation (BMV), that assist neonates in adapting to extrauterine life [8]. Timely application of simple resuscitation measures at birth and within the first hour of life could save as many as two-thirds of neonates [9].

For research to be clinically relevant, the clinical environment must be considered. In areas with limited clinical resources, such as the United Republic of Tanzania (Tanzania), significant challenges, including the high number of resuscitations performed, must be addressed [10]. Midwives manage the majority of deliveries in the labor ward at the research site (40-80 deliveries per day), which includes complex obstetric and neonatal emergencies in the labor ward on a daily basis. Examples of conditions encountered by these midwives include eclampsia, placental abruption, postpartum hemorrhage, breech presentation, multiple pregnancy (twins), perineal suturing, and vacuum deliveries for prolonged and obstructed labor [11].

Midwives have limited assistance from obstetricians, who are also significantly short-staffed, and manage highly dynamic priorities [12]. Doctors are rarely involved in neonatal resuscitation at delivery [6].

Intrapartum-related hypoxia, which can lead to birth asphyxia, can be caused by many factors, such as prolonged and obstructed labor, underlying conditions resulting in poor fetal growth (such as maternal anemia, maternal human immunodeficiency virus (HIV) infection, or poor maternal nutrition and health), eclampsia and placental abruption [6]. These factors are exacerbated by limited resources, such as a lack of fetal heart rate monitors, basic medications, running water, soap and gloves for infection prevention [13, 14]. The 2016 WHO [15] report on “Midwives Voices Midwives Realities” stated that 53% of African midwives value “being listened to” [16]. Midwives’ stories are key to understanding their experience, and engagement, listening, interpretation, and understanding can lead to a dialectical relationship between the researcher and research subject [17].

The aim of this study was to listen to and understand the stories, beliefs, attitudes, and experiences of Tanzanian midwives to reveal solutions and identify factors that influence successful neonatal resuscitation to inform clinical practice and reduce the incidence of VEND [14, 18].

## Methods

### Study design

This was a qualitative narrative inquiry study utilizing the 32-item consolidated criteria for reporting qualitative research (COREQ) checklist [19]. Narrative inquiry “amplifies voices that may have otherwise remained silent” [20, 21]. Audio-recorded, semistructured individual interviews with midwives were conducted. This method was utilized because it highlights midwives’ stories and identifies noteworthy factors and solutions. “Stories can make nursing practice visible” [22], stories are capable of impacting organizational understanding and stories can be a catalyst for change [23]. Listening to stories can assist midwives in decision making, support learning [24], and help develop resilience [25].

### Participants

The research participants were midwives working in the labor ward of a hospital located on the eastern coast of Tanzania in the city of Dar es Salaam. The hospital serves an area with a population of approximately 1.5 million people [26]. Approximately 40–80 deliveries are performed per day in the labor ward (13,618 in 2019). The training of the midwives interviewed ranged from 1 to 2 years (enrolled nurse midwives) to 2-3 years (registered nurse midwives), and one midwife held a master’s degree in midwifery.

### Inclusion criteria and exclusion criteria

The inclusion criteria were midwives aged over 18 years old who had worked in the labor and/or early-labor wards for a minimum of 6 months, managed a minimum of three neonatal resuscitations and witnessed or experienced managing a VEND while on shift.

These criteria were set to ensure that participants had relevant experience on the topic of this study.

### Ethical considerations

Ethical approval was obtained from the research site and university. Local approval was acquired from the director, Matron, and Charge Sister of the facility.

### Sample size and participants

A poster explaining the research and inviting midwives on the labor ward to participate was put up in the wards a few days prior to study commencement. The labor ward has a permanent staff of 13 nurse midwives. The participants volunteered to be part of the study and did not receive any money or benefit from inclusion in the study.

### Data collection

The data collection period was July to September 2019. A private training room was provided for confidential interviews. Each midwife was interviewed twice on separate days at times that were convenient for the midwives. Informed consent was obtained in writing on the day of the interviews after all volunteers were given comprehensive written information about the project in both Kiswahili and English. Interviews were conducted in English. All interviewed midwives were bilingual. Interviews were conducted by JB, a female interviewer and senior clinical midwife, and another female researcher midwife, CB, was present.

An interview guide with questions related to the midwife’s role in labor and delivery, especially neonatal resuscitation, and factors impacting successful resuscitation and quality of care (especially when it resulted in VEND) was used. For the purpose of this study, neonatal resuscitation was defined as resuscitation of a baby not breathing at birth. After each interview, the interviewer wrote notes to assist in clarifying points made by the participant and to capture any nuances, pauses, body language or emotions unable to be transcribed or audio recorded [27].

The initial interviews with the eight midwives lasted 40–60 min. After each initial interview, the interviewer, JB, and the other registered midwife researcher, CB, reviewed the audio recordings and interview notes to address additional questions and seek clarification on certain points, as well as to recognize when no new information had emerged [28]. The second interview lasted between 25 and 40 min. Data collection ceased at the point of data saturation, which was the point when no new significant information arose [29] and no new themes appeared [30]. Other midwives, although willing to be interviewed, were unable to participate due to work schedules, travel limitations or staffing constraints preventing time away from the ward.

### Data analysis

Audio recordings were transcribed verbatim. If any Kiswahili words were spoken in the interviews, a timestamp was placed, and a midwife (who holds a Master’s degree in midwifery research) was asked to translate the words. Transcripts were checked by JB and CB against the original recordings to correct any inaccuracies in transcription. All data (mp4 audio files, typed transcriptions of the audio files and saved transcripts) were deidentified to ensure privacy and confidentiality of the participants and stored on secure password-protected drives.

Thematic analysis was guided by the stages defined by Braun et al. [31] and began with five to six initial run-throughs of each audio file and transcript by JB and CB for familiarization and identification of embryonic themes [21]. The analysis of the data was performed by methodically reading, noting, categorizing, construing key data, and arranging key phrases and similar leitmotifs, with substantial deliberation on dominant themes [30, 32]. The coding and definition of themes were completed by three researchers (JB, CB, JM) and verified by other authors. All three coders agreed that data saturation had been reached [30]. By the 6th interview, the same themes emerged, which was further confirmed in interviews 7 and 8. A final consensus was reached by all authors after many iterations.

### Reflexivity

Reflexivity is defined as the ability to understand that the researcher is both an insider (practitioner) and an outsider (observer) at the same time [33].

Reflexivity was supported by acknowledging the perceived assumptions and clinical experiences of JB, CB, and JM in this research setting [34].

### Rigor

The data collection and analysis techniques contributed to the trustworthiness of the data [35]. The convergent process of multiple researchers systematically and cyclically probing the data and refining the themes added to the rigor [35]. Member checking or “participant validation” of the data enhanced trustworthiness and validity, thus ensuring that the conclusions, findings, and intuitions were dependable [36] and resonated with the stories’ meanings and authentic experiences shared by the midwives [37, 38].

Interviews commenced with an open-ended question using an interview guide in both English and Kiswahili and were carried out by the same two investigators to ensure the reliability of the midwives’ narratives [39]. After each interview, discussions were held between JB and CB, and the interview guide questions were adapted to account for previous interview data, elicit further elucidation of their already intense stories told and to allow the midwives to voice their experiences more spontaneously [40].

All midwives were interviewed twice to clarify and ensure that the researchers had captured their stories accurately and to delve into the evolving themes from the first interviews. Additionally, it also allowed the midwives to share any further stories they felt that they wished to share. To enhance confirmability, this study research group carried out many peer reviews and debriefing sessions, ensuring the trustworthiness of the stories.

The midwives and obstetrician had extended, focused interactions with the participants regarding neonatal resuscitation and VEND experiences at this research site, which enhanced the study credibility [39]. Detailed descriptions of the methodology, the narrative design, and the context of the setting may enhance transferability [41].

## Results

Eight midwives (seven females, one male; aged between 28 and 52) with an average of 13.5 years of professional midwifery experience/practice (ranging from 4 to 25 years of experience) participated in the study (see Table 1). All had performed over 200 neonatal resuscitations in a labor ward, with an average of 825 neonatal resuscitations carried out by each midwife. Each midwife was interviewed twice.

**Table 1:** A summary of the characteristics of the midwives interviewed

Average range of ages	Educational background	Experience managing neonatal resuscitation	Years in labor ward
28–54	• 2-9 years training on average • Master’s of Midwifery and Women’s Health; 9 years training • 6 Registered nurse/midwife (RNM); 4 years training • 7 Enrolled nurse/midwife (ENM); 2 years training	• 100-2500 neonatal resuscitations performed by each participant • 825 neonatal resuscitations carried out by each midwife on average	• 13.5 years on average • Range 4-25 years

### Themes

The study identified three dominant themes that influenced effective neonatal resuscitation.

Each dominant theme is described in detail below (see Additional file 4 Pictorial Themes).

#### Theme 1: hands-on training (“HOT”) with clinical support (mentorship) during actual emergency neonatal resuscitation events decreases fear and enables clinical skills transfer

A recurring element in interviews was that midwives focused on saving the neonates with *skilled* intervention. “HOT” in resuscitation was highlighted as a significant element in successful skills transfer. The midwives articulated that the knowledge that they received while standing beside their colleagues and receiving real-time instruction was a vital part of their training. They stressed that mentorship was best achieved through active, clinical skills support with guided troubleshooting and hands-on training rather than through esoteric support or classroom training.

Furthermore, some midwives expressed that fear and a lack of confidence impeded their learning. One midwife recalled her amazement upon first witnessing that BMV worked, with the event persuading and enabling her to change her beliefs and clinical practice and teach with conviction. The confidence of mentors is crucial for mentees to gain confidence and overcome fear.

Midwives experienced “HOT” resuscitation in the labor ward after a short simulated session (with a mannikin) as a powerful medium for change because of the power of vicarious learning. This approach is proven to be successful in imparting resuscitation skills and techniques to other midwives:

*“I think demonstration, demonstration. If every shift, before starting the activities of the day, practiced resuscitation first, before every shift. You come in the morning, after cleaning and preparing the equipment, and you start with resuscitation.*

*The main problem of the labor ward – ‘resuscitation’. If everyone knows how to resuscitate, death will be rare” (Mkunga_06)*

All midwives recognized and expressed that “HOT” resuscitation by Champion midwives (those highly skilled in neonatal resuscitation) was useful for teaching other midwives updated (new) skills, as well as correcting old habits and out-of-date techniques. Midwives considered teaching an integral part of their role. All expressed that training, learning, and absorbing accurate neonatal resuscitation skills take time*.*

One byproduct of mentoring was the emergence of leadership qualities and pride in teamwork among midwives. Emergent collective self-efficacy created a propensity to be more actively engaged and forthright in correcting incorrect actions of colleagues and guiding them in the correct actions because of the perceived successes in reducing VEND and fresh stillbirth (FSB).

Some midwives expressed a desire to be more actively involved in weekly multidisciplinary perinatal clinical meetings to discuss and learn from neonatal mortality and morbidities and carry out training:

*“As a midwife, it's easy to help others if we are sitting in a meeting, a regular meeting. For the meeting on wellness, we talk about how to care for the patient in the ward. Regular and monthly meetings of the staff of the labor ward are held, reminding the midwife of her roles, how to help the mother, how to help the baby so that they begin and end labor safely with their baby.” Mkunga_06*

##### “HOT” in resuscitation builds confidence and changes beliefs and behaviors

Successful live resuscitations built confidence, especially when midwives-in-training saw BMV resulting in a well oxygenated neonate (pink skin tone) and better outcomes than a penguin suction device only:

*“Live practice is better, without using a doll. A doll is easy. We take this, ‘Ah, this doll!’ But when you see a live baby, a real baby … Because the dummy doesn’t give confidence. It teaches a skill” (Mkunga_08)*

Midwives expressed that confidence allowed them to perform skilled resuscitation on birth-asphyxiated neonates and obtain better outcomes. Initial fear and lack of confidence were cited by midwives as constraining and discouraging of their learning updated neonatal resuscitation skills. Additionally, starting resuscitation without a penguin bulb suction device is contrary to their initial nursing and midwifery training. Witnessing and/or partaking in successful resuscitations directly (hands-on with full peer support) built confidence, especially when midwives observed superior results and outcomes of BMV.

Fear and anxiety are reduced when midwives observe successful outcomes in the labor ward.

*“Sometimes they are scared. Yeah. I’m afraid. When you call, ‘you have to do Helping Babies Breathe - HBB!’ I’m running. I run away when I hear that” (Mkunga_08)*

##### Seeing positive physiological changes firsthand was helpful

The delivery of air via effective BMV results in visible changes in a neonate’s color, heart rate and respiration. Having a mentor and being able to see the physiological changes in neonates following BMV was cited as particularly helpful. Doing so allows midwives to gain confidence in their ability to assess skin color changes, neonatal tone and activity, the breathing pattern or lack thereof during resuscitation and heart rate:

*“I’ve now seen how a baby changes, and I am much more successful … use air and the baby gets pink. The success makes me believe in HBB.*

*Before we used to use penguins, and the baby would die of birth asphyxia.*

*We didn’t see the change.*

*A pink baby gives me hope and a sign of my success” (Mkunga_08)*

#### Theme 2: unequivocal commitment to the Golden Minute^©^ and work mindset of the midwife

Midwives are committed and dedicated to saving mothers and babies, and they consider midwifery care to be from the “heart.” The midwives articulated that a mother carries her baby for 9 months and that a midwife must be committed to looking after the mother for 1 day and the baby for one Golden Minute. They stated that their job requires “heart” and that money was not their chief motivator.

It was apparent in the data that many Tanzanian midwives felt the approach to their role and that the mindset in their job was equally important. The overwhelming consensus was that there is an intrinsic belief that the midwife’s mind must be focused, ever present, and actively willing to learn and adapt to the wards’ clinical load.

*“If you do [midwifery] like ‘a job,’ you are not going to work properly, because you are working because of money, not because you are assisting the helpless mothers. And this is not from the heart. You are not going to be a good midwife if you do it like that” (Mkunga_03)*

Some midwives were explicit that saving babies and preventing VEND is *more* than skill and training; some articulated this with passion in their voices, cadence, tone, and hand gestures during interviews:

*“I think a midwife needs heart, needs heart, because if you have no heart and no real, real internal calling to be a midwife, it is hard to work. We must try to work hard, hard, hard and save all those mothers” (Mkunga_02)*

##### Monitoring labor progress and fetal heart rate

Key points in this theme are intrapartum preparation with a focus on quality care for neonates and predicting and preventing VEND. Preparing for delivery meant removing the suction penguin devices and instead preparing bags and masks. Vigilantly monitoring key labor parameters (e.g., fetal heart rate and maternal factors in prolonged or obstructed labor or a prolonged second stage that can significantly impact the fetus) influences clinicians’ decisions regarding care. These decisions may impact the timing and level of interventions that may expedite delivery and therefore prevent VEND, such as vacuum delivery or cesarean section.

##### Preparation for every delivery

Half of the interviewed midwives spoke of the importance of being prepared for neonatal resuscitation at *every* delivery by monitoring the mother’s wellbeing in regard to the progress of labor and fetal wellbeing. Anticipating neonatal resuscitation at delivery could be due to nonreassuring fetal heart rates, placental abruption, or an eclamptic mother and allows a midwife to better predict and manage whether the need for resuscitation is imminent.

One midwife stated the following:

*“That’s why when you have a patient, you know the type of baby that might be delivered, so you prepare. The mother needs a caesarean, you prepare for a caesarean, communicate it to the theatre, to the doctor. If you follow our midwife skills, then fresh stillbirth will be history. But fresh stillbirth will not be history because of neglect” (Mkunga_06)*

Therefore, preparing the bag and mask and being prepared to carry out skilled resuscitation is important:

*“And no delay because the mtoto (*‘*mtoto’ means ‘baby’ in Swahili) is there, the champion is there, the neonatologist is there, and the equipment is there. You are supposed to stay with the bag and mask and penguin beside your bed” (Mkunga_01).*

Preparation of the birth area and resuscitation equipment allows for a timely response at delivery if resuscitation is required. Valuable time is lost when running to another room to find a clean bag and mask.

Another midwife expressed the importance of preparation in many aspects of labor and delivery:

*“What’s the baby need? Who is the mother who has come into the labor ward? What are the needs of this mother? Don’t know. Observe the mother. What’s their need? If you know their need, help accordingly. If this mother needs some help with the fetal heart now, warn the fetal heart specialists. If you need to take vital signs now, take vital signs. If this mother needs to be re-examined, perform the re-examination. Now this mother is in the second stage, what’s the need? Prepare, they will be fine” (Mkunga_06)*

##### Infection prevention

Preventing sepsis by ensuring preparation of a clean environment was emphasized. Midwives cited that infection prevention and timeliness of ventilation is better achieved when neonatal resuscitation is carried out on the mother’s chest rather than on the baby bench, which has a higher cross-contamination and infection risk, wastes precious time and creates undo fluster to cut the cord early and run to the baby bench.

There is a bench in the labor ward that can accommodate up to ten babies wrapped in kangas (“kangas” (plural) is a Swahili term for light cotton fabric that is usually printed with colored designs and mainly used for women’s clothing).

Mothers, in many cases, cannot hold on to their babies while having perineal repair or other management postdelivery. This bench creates cross-infection risks for all babies, which was expressed by some midwives.

“*But the place for HBB is too small, you see. Sometimes you put almost 10 babies there, and in the same place, we have to do HBB, so it is not suitable. It is not enough.*

*I think that place can cause cross-infection because you find one baby sucking on another baby, sucking on its hand, on the clothes of the other baby. I don’t like that place. Better with the mother” (Mkunga_05)*

##### Starting resuscitation on the mother’s chest

Starting neonatal resuscitation at the mother’s bedside or on the mother’s chest was deemed to expedite resuscitation and assist in infection prevention and thermoregulation for the baby. If midwives are alone or have limited staff (e.g., night duty), the mother can watch the neonate while the midwife attends to the third stage of labor and to any further treatment for the mother (e.g., postpartum hemorrhage (PPH), perineal sutures). Most importantly, the Golden Minute is misused, with unnecessary delays such as cutting the cord and moving to another location. Instead, resuscitation can be started immediately at the bedside.

The Golden Minute is a critical time period for assisting neonates in taking their first breath. This period is thwarted when no *clean* resuscitation equipment is ready by the bedside or near labor ward delivery beds. This can be due to large numbers of resuscitations and the timeframe needed to decontaminate bag and mask equipment outweighing the resuscitation workload. The unproductive time spent seeking out a clean bag and mask was highlighted by midwives:

*“Sometimes you deliver a baby who needs resuscitation, and you have no equipment for resuscitation nearby, so you must run to find the equipment, and the resuscitation needs to be performed in the golden minute, one minute to help the baby to breathe. So, it can take more than one minute before you can help the baby, and this is very, very discouraging to me” (Mkunga_2)*

#### Theme 3: strategies to reduce barriers to successful resuscitation, especially unnecessary suctioning as the first response

##### Proclivity to perform suction first consistently delays ventilation; instead, “air air air” should be first

The proclivity of midwives to start all resuscitations with suctioning of the neonate’s oropharynx using a bulb suction device called a penguin causes critical delays in ventilation.

This dogmatic clinical behavior is one of the single most difficult barriers to overcome and is a major impediment when teaching BMV after basic stimulation of the neonate. Midwives assess the neonates when first born for heart rate, skin color, activity, and breathing or lack of breathing. Immediate steps are drying of the neonates to facilitate thermoregulation and stimulating breathing in neonates who fail to breathe on their own at birth.

The mantra of “*air air air”* is reiterated to midwives learning resuscitation.

*“Establishing a lot of champions on each shift who can insist on ‘don’t suck; air first, don’t suck; air first’ and you can promote air. The air you introduce to the baby can be very good” (Mkunga_01)*

Furthermore, due to the lack of sufficient equipment, the time consumption required by manual decontamination and cleaning and midwives being “time poor,” there are not always clean penguin suction devices or bags and masks available for resuscitations, which increases the risk of infection. Some midwives recounted instances of colleagues using used/nonsterile penguin suction devices with the (incorrect) rationale that secretion removal is far more important than preventing infection or starting BMV.

One midwife expressed a simple lesson she gave to midwives in training:

*“The penguin. Put it aside … Ambu bag, Ambu bag, Ambu bag, air, air, air—yes, good” (Mkunga_08).*

Midwives expressed frustration at colleagues who were slow to act with a bag and mask and instead used the penguin in the first instance during an emergency. Furthermore, the midwives recognized that there are times, rarely, when suctioning *is* required for tacky, thick, tenacious secretions.

Midwives stated that their midwifery course taught them to use the penguin suction device first. Only after this are they introduced to BMV, which involves a significant change in skills and requires learning new material:

*“And you cannot, with a penguin, suck up the lungs. No. A baby just needs air, to breathe as a human being. If the baby is breathing, I think you will save the baby. Let’s just try. Removing penguins from the labor ward can help the baby to begin breathing. Maybe because they need air. Human beings, what do they need? ‘Air’” (Mkunga_06)*

##### Scarcity of skilled staff, basic medications, equipment, and clinical resources

Barriers to successful neonatal resuscitation for birth-asphyxiated neonates suggested by midwives included a lack of basic resources such as gloves, medication and equipment (e.g., urinary catheters) required for cesarean section operations, postpartum hemorrhage, eclampsia, low morale, poor compensation, and uneducated mothers with poor antenatal care leading to complications and delays in coming to the hospital, which thwarts timely care, and the use of tribal herbal medicine, which can cause precipitous labor with problems such as a ruptured uterus. Furthermore, running water and 24/7 electricity are not reliably available.

An environment with a regular lack of resources was identified as having a negative impact on performance and morale:

*“If I don’t get enough resources, I feel bad because my work will be hindered. I will not be able to perform my work well. So, when I have enough resources, I can perform my work effectively” (Mkunga_03)*

##### Overcrowded, underresourced environment

Fundamental requirements for a medical facility, such as reliable running water, soap, and sterile clothes with which to dry hands, are mostly unavailable at this research hospital. The labor ward is an overcrowded environment. The facility lacks supplies of all types, including gloves, intravenous fluids, saline, syringes, cord clamps, artery forceps, indwelling urinary catheters and lifesaving medications such as magnesium sulfate, and there is limited blood to manage ante- and postpartum bleeding. These shortages render basic infection control and management of complex cases incredibly difficult, which can cascade into poor neonatal outcomes.

The lack of basic medical resources means midwives are unable to protect themselves during labor and delivery, which is an impediment to being able to manage the delivery for any mother, especially for those who pose a greater infection risk (e.g., mothers who are HIV or coronavirus disease 2019 (COVID-19) positive).

Complex and complicated deliveries are all managed in the same labor ward as uncomplicated deliveries. Cases such as eclampsia-related emergencies, complex deliveries such as breech presentations and twins, and neonatal and maternal resuscitations at delivery were not moved to a separate ward.

The majority of midwives emphasized that the overwhelming workload and low staff-to-patient ratio are major problems and barriers to safe and effective skilled resuscitation, especially at night when there may only be two staff members on duty. Intermittent electricity and a lack of torches further worsen the night-time situation. More midwives are needed simply to manage the number of normal deliveries, but there is also a desperate need for skilled, experienced midwives who are clinically competent at neonatal resuscitations.

##### Skilled staff rotated out of the labor ward

Staff at the research hospital are frequently rotated in and out of the labor ward, so a midwife or nurse who has just joined the labor ward may be unskilled in neonatal resuscitation, which can be a major hindrance to providing effective skilled care for both mothers and neonates. Furthermore, shortly after neonatal resuscitation training, they may be rotated to another ward:

*“The midwife in the labor ward is not a permanent nurse at the site. They’ll come, you train the midwife to understand the labor ward, then three months later they go to another unit, and another midwife comes who is not as skillful” (Mkunga_06)*

The research hospital handles a minimum of 40–80 deliveries daily (sometimes up to 100).

The research hospital has insufficient staff to manage even normal labor and deliveries, which are commonly complicated by very sick mothers with multifaceted problems outside of pregnancy, such as, HIV positivity, anemia, malnutrition, undiagnosed twins, and breech presentation. Furthermore, the use of traditional herbs can create precipitous, strong unpredictable labor leading to uterine rupture, especially in mothers with previous scarring from cesarean section. Lack of staff also decreases the quality of intrapartum and postpartum care for mothers. Furthermore, neonates may be severely compromised, requiring priority care. These midwives face a dilemma between fulfilling the role of a midwife and facing the unconscionable quandary of having to choose who receives care.

## Discussion

It was evident from the midwife’s impassioned narratives that they welcomed an opportunity to be part of the solution to reduce VEND. The midwives’ stories lend an authentic voice that validates and reveals the *humanness* of their experience [42, 43]. Many studies on VEND and fresh still birth in countries with limited resources over the last decade have been *focused on* the midwives, rather than *conducted by* the midwives. WHO (Page 2) stated midwives “*express how they are hindered through a lack of voice in creating the change and delivering the creative solutions they know are so badly needed*” [15].

Previous studies have emphasized some of the barriers to improving neonatal survival in low-resource settings, such as staff shortages and the lack of equipment [44], yet few have addressed successful solutions and insights that midwives provide directly through narratives to overcome the lack of sustainable clinical skills transfer for efficiently resuscitating a nonbreathing baby at birth.

The participants were involved in a custom abridged training program based on the Helping Babies Breathe (HBB®) program, which was called the Champion program. Champion midwives completed the HBB workbook, which was available in English or Kiswahili, as well as an additional twenty-question written HBB exam in their own time. Each midwife received guided simulated practice in BMV skills using a mannikin in either the labor ward or the training clinic. The difference in this program compared to previous HBB implementations is that the Champion program includes an additional critical element of “HOT” (hands-on training) in live neonatal resuscitation and mentoring in the labor ward [45].

All midwives who received neonatal resuscitation training read aloud a pledge, signed it, and hung it on the wall of the clinic (see Additional file 3). The design, words and intent of the pledge developed by two authors (JB and CB) was to focus midwives’ attention on striving for a higher level of compassionate professional care and collective teamwork, including the overall mission as midwives in service to the mothers and babies of Tanzania. The pledge aids in instilling pride, behavioral change, and hope in the midwives (see Additional file 3) [46].

This instruction was initially provided by two researchers (JB and CB). Some local midwives have achieved mastery, earning the title of “Champion”.

This study complements and addresses the recommendation in the qualitative study carried out at a rural community hospital in the southwestern area of the Mbulu district [6]. The study recommends interviewing midwives in an urban setting to provide more “insights and enrich the discussion” [6, 47].

### Beliefs

A dominant theme was midwives’ beliefs regarding what makes a “good” midwife—a disposition of kindness, caring and compassion; skillfulness; knowledge; and being “present” for mothers. Notably, despite the overwhelming workload, high rates of VEND and other significant barriers, the midwives’ commitment to their position came “from the heart,” with an unrelenting focus on providing skilled care and obtaining the outcome of interest—a live baby. Despite sometimes feeling demoralized and frustrated by certain colleagues’ lack of provision of skilled care and commitment, the midwives remained resolved in the pursuit of teaching, mentoring and building confidence in their colleagues as part of their professional role (see Additional file 2). Similar to other studies that identified the despair and pain that midwives associate with high rates of VEND, the midwives continued to be committed to collaborating as a team with the expressed hope of reducing VEND rates [48].

Narratives give meaning and context and are not static; however, they do represent the “lived” world at a given time, which validates the very values and messages conveyed by the midwives [49]. At times, there was a sense of weariness and sadness as the midwives shared their stories, yet in the telling their stories, practical solutions, intuitive beliefs and refreshing insights on how to overcome VEND were added to the narratives [50, 51]. Storytelling was also a safe place for midwives to talk through harrowing experiences, voice disappointments in colleagues and vent frustrations due to the lack of resources to carry out good midwifery care. This can be a cathartic experience that can also aid the midwives in finding meaning and a way forward to embrace new initiatives, a self-healing for the soul, (Page 126) [52].

### Environment

The respondents were frustrated by the constant barriers in their working environment and changes to staff and the subsequent need to train new staff, some of whom did not want to learn the bag and mask skills and tended to gravitate toward working the night shift to avoid training. Some midwives believed that certain nonengaged staff members should be moved from the labor ward to another part of the hospital, thereby leaving the midwives who are committed and valued their professional skills and role in caring for mothers and babies. The selection of skilled midwives in this setting is thus an important consideration, as all midwives need to be skilled in neonatal resuscitation due to limited staff and overwhelming caseloads [14].

Urban and smaller regional hospitals in more remote regions may have even more difficulty attaining and keeping skilled staff; moreover, the availability of working bag and mask resuscitation equipment is often poor [14]. Mothers in farming and remote rural areas may have more intensive barriers to overcome in terms of having access to timely transport to the hospital, which could impact neonatal outcomes [53]. Midwives’ stories are similar in highlighting challenges of fear and lack of confidence, overwhelming workloads and an overwhelming patient-to-staff ratio [6].

A commitment by hospital procurement to provide additional working bags and masks to facilities along with a reorganization of workflows for bag and mask cleaning and sterilization may assist in overcoming barriers to successful resuscitation [54].

### Behavior

Bandura’s [55] social cognitive theory was applied to the final dominant themes and found to be consistent with three elements: beliefs, behaviors and environment. This triadic relationship was explored and revealed, as midwives told their stories and described through the elements that impacted their behavior even under such challenging conditions.

Moreover, the midwives’ narratives emphasized various levels of self-efficacy and collective self-efficacy. The midwives’ words elucidated their own varying levels of confidence in managing neonatal resuscitations, as they reflected on how they changed and adapted to situations as they arose. This manifests Bandura’s [55] theory of mastery and self-efficacy. Some participants’ degrees of control of or dedication to successful neonatal resuscitation showed high levels of self-efficacy.

The desire for mentorship and supportive team dynamics articulated by the midwives highlights the implications of linking individuals into a “collective,” thus empowering the midwives to collectively find actionable solutions [56, 57].

Emergent “collective” self-efficacy created a propensity to be more actively engaged and forthright in correcting a colleague’s incorrect actions and guiding them in skilled actions because of the perceived successes in reducing VEND and FSB. The power of “collective” self-efficacy to facilitate behavioral change is especially critical in guiding midwives to unlearn the ingrained first response to perform suctioning and focus on the Golden Minute at the mother’s bedside.

A group focused on successful resuscitation practices creates a force that can affect change and obtain positive outcomes for neonates through “collective” self-efficacy [55, 58].

A sense of urgency is paramount in the event of a nonbreathing neonate [59], as their chance of morbidity increases by 16% for every 30 s of delay in starting ventilation [60].

The results were presented to the participant midwives, charge sister of the labor ward and matron, and the results generated discussion and encouraged further insights.

For example, along with being prepared for any delivery to require resuscitation, midwives articulated that the dynamic clinically relevant knowledge that they received while standing beside their colleagues and state that receiving real-time mentorship was a vital part of their training.

Some studies on midwives in sub-Saharan Africa have highlighted a demoralized workforce [48, 61, 62], yet despite being disheartened, their focus on a live mother and baby through heartfelt care resonated within these midwives’ stories [50].

Finally, the midwives’ narratives provide hope and vision for the future [63], and the midwives’ descriptions of their experiences in hauntingly evocative stories resonate, if only we will listen in the silence [64, 65].

### Limitations

The study was limited by the midwives being selected from an urban hospital setting only. Replication of this research in other settings (e.g., other hospitals from regional referral or local district hospitals in different districts and regions of Tanzania, both rural and urban) would help confirm the transferability of the results. The midwives interviewed had received neonatal resuscitation training through the Champion program, which could bias the findings because these midwives had received training and seen the benefits of the new skills before they participated in the study, potentially skewing them toward being advocates for training and change. However, the Champion program is only one factor influencing the experiences and insights of the midwives regarding neonatal resuscitation and VEND; thus, it does not impact all the narratives.

The two main researchers have worked in this labor ward over 5 years and could thus be deemed “insiders,” which could have shaped the midwives’ narratives. Furthermore, the researcher’s thematic elucidations (JB, CB, JM) may have been influenced by their own experiences. Thorough discussion, peer review and debriefing by experienced researchers with nonclinical input were used to address this issue. However, the “insider” relationships forged over a number of years with the midwives created data richness within a shorter timeframe, as the midwives were comfortable with freely sharing their stories, feelings, insights and experiences because they knew that the researchers were familiar with their working environment and were impacted by similar experiences.

### Future research

Further study with midwives in other settings and/or who have not received Champion training is required to determine whether champion training influences experiences and insights. Additionally, research on the champion midwives who are now skilled mentors and their steps in progress with regard to behavioral learning has influenced their neonatal resuscitation skills. Serious gaming is an emerging field that captures real-life problems [66]. Solutions developed through decision-making and gaming can have a positive effect on clinical practice [66]. Research that evaluates a simple serious game that highlights basic key neonatal resuscitation skills and its effect in Tanzania would also be of value. The impact of providing a supportive directed orientation/mentor allocation to new staff in the labor ward could assist in developing a pool of champions who may be able to take the key elements of the training to outside clinics, with research on the flow-on effect of the outcomes of mothers and babies in regard to VEND. Finally, one champion midwife suggested that suction devices be removed from the labor ward as a standard and researched the impact of VEND and resuscitation outcomes with only essential warm, dry stimulation and bag and mask ventilation.

## Conclusions

There is a dearth of studies on the narrative of midwives in clinical settings with limited resources, especially in the context of VEND due to poor clinical competency within an overwhelmed labor ward. The voices of midwifes in sub-Saharan Africa are rarely heard. Moreover, their stories have significantly added to practical insightful solutions, exposed beliefs and impacted clinical practice in a positive way, both individually and collectively. Neonatal resuscitation in areas with limited clinical resources is both simple and complex. Simple key interventions such as warming, drying and stimulating a baby will save many babies who are born not breathing [67]. The complexity comes in the management of multiple extrinsic factors by midwives that are not encountered in developed countries. Not all babies can be saved by simple bag and mask methods [68]. Ingrained habits hinder and delay successful resuscitation; therefore, unlearning old practices is vital to learning new skills and mastering the bag and mask skills involved in administering “air air air [45].

Despite the barriers, the midwife stories highlight many influences, both positive and negative, that impact successful resuscitation. Bandura’s social cognitive theory resonated in the interviews and highlighted the dynamic triad of reciprocating elements, the interplay between beliefs, environment and behaviors, showing that positive change can happen slowly. Training with a focus on realistic, culturally relevant interventions, with a strong sense of collective self-efficacy, emerged as a biproduct of mentorship and skilled midwives, called Champions. Bringing this training into the national midwifery and nursing curriculum could enhance clinical skills and update newer contemporary practices in neonatal resuscitation. Such training can create inspiration and hope for other midwives who confront VEND almost daily in these settings.

## Supplementary Information

**Additional file 1.** Relevance to clinical practice in limited resourced countries.

**Additional file 2.** Impact on the wider global clinical midwifery community.

**Additional file 3.** Midwife Vision Pledge.

**Additional file 4.** Pictorial Themes.

## Data Availability

The data that support the findings of this study are available from the National Institute for Medical Research, Tanzania, but restrictions apply to the availability of these data, so they are not publicly available. Data are, however, available from the authors upon reasonable request and with permission from the National Institute for Medical Research, Tanzania.

## Abbreviations

BMV: Bag and mask ventilation
FSB: Fresh stillbirth
HBB: Helping Babies Breathe
HIV: Human immunodeficiency virus
VEND: Very early neonatal death
HOT: Hands-on-training during real live resuscitation in a nonbreathing neonate
