# Supplementary material for: Silent voices of the midwives: factors that influence midwives’ achievement of successful neonatal resuscitation in sub-Saharan Africa: a narrative inquiry
Source: BMC Pregnancy Childbirth. 2022 Jan 16;22:39. doi: 10.1186/s12884-021-04339-7 (PMC8761383; doi:10.1186/s12884-021-04339-7)
Supplement: Supplementary file 1 — Additional file 1. Relevance to clinical practice in limited resourced countries. [file 12884_2021_4339_MOESM1_ESM.docx]

**Additional file 1: Relevance to clinical practice in limited resourced countries**

1. An unwavering focused commitment to the neonate for at least one minute immediately after birth. *Time is vital, and air is cardinal.* Delays in either can cascades a neonatal into almost irreversible secondary apnea.
2. Midwives must *un*learn outdated practices (e.g., deep long suctioning) and see firsthand positive visible changes in the neonate’s color, breathing and tone to appreciate the significance of ‘*air air air*’ over suction as the critical first line intervention. Mentoring HOT resuscitation at the beside highlights the key elements of successful resuscitation.
3. Vigilantly intrapartum monitoring of the mother and the fetus can assist a midwife in anticipating a neonatal resuscitation so that s/he is prepared with bag and mask for *every* delivery in order to start timely skilled interventions (warm dry stimulate, then if required bag and mask). Start the resuscitation immediately at the bedside, as it saves precious time.
4. Short bursts (5-10 Minutes) of bag and mask ventilation training sessions held often (2-3 times a week) *in* labor ward on a plastic mannikin, can increase confidence, decrease anxiety, and build self-efficacy. Moreover, the training is to be fully supportive, without rebuke, to create a sense of empowerment, by vicarious learning.
5. ‘HOT’ Resus’ bedside training and daily mentoring is an essential element to build skills, confidence, and mastery of resuscitation competencies.
6. Keep skilled champion staff in labor ward. Rotation of skilled staff *out of labor ward* is demoralizing and dilutes the skills required for successful neonatal resuscitation in this dynamic fast paced ward. Mentor attitudes of care, professionalism, team-oriented approaches, and a willingness to learn and mentor others. If they attitudinally see it as only a job, then the VEND numbers will be slower to change and their skills could be utilized in other wards.
