# Supplementary material for: Silent voices of the midwives: factors that influence midwives’ achievement of successful neonatal resuscitation in sub-Saharan Africa: a narrative inquiry
Source: BMC Pregnancy Childbirth. 2022 Jan 16;22:39. doi: 10.1186/s12884-021-04339-7 (PMC8761383; doi:10.1186/s12884-021-04339-7)
Supplement: Supplementary file 2 — Additional file 2. Impact on the wider global clinical midwifery community. [file 12884_2021_4339_MOESM2_ESM.docx]

**Additional file 2: Impact on the wider global clinical midwifery community**

1. The voices of midwives in areas with limited clinical resources are rarely heard, yet these practitioners highlight solutions and insights for the management of overwhelming odds while remaining focused on reducing numbers of very early neonatal deaths.
2. Training in interventions for neonatal resuscitation in a clinical setting with limited resources was adapted to overcome the limitations of simulated training alone. A mentored, real-time “HOT” resuscitation champion program was developed to build confidence and mastery in bag-and-mask ventilation and increase hope.
3. Midwives in limited clinically resourced settings continue to strive to learn, adapt and impact the mothers and babies they care for and in turn inspire their colleagues to learn and support each other despite engulfing patient loads with too few basic supplies.
