# Supplementary material for: Silent voices of the midwives: factors that influence midwives’ achievement of successful neonatal resuscitation in sub-Saharan Africa: a narrative inquiry
Source: BMC Pregnancy Childbirth. 2022 Jan 16;22:39. doi: 10.1186/s12884-021-04339-7 (PMC8761383; doi:10.1186/s12884-021-04339-7)
Supplement: Supplementary file 3 — Additional file 3. Midwife Vision Pledge. [file 12884_2021_4339_MOESM3_ESM.docx]

# Additional file 3: Midwife Vision Pledge

Table 0‑1 Midwife Vision Pledge in English

| **Midwife Vision Pledge**  I pledge to hold myself to the highest professional clinical standards, care and conduct.  I will at all times be kind, compassionate and focused on the best outcomes for the mothers and the babies under my care.  I acknowledge I am part of a team and will support my fellow Midwives, Nurses and Professional clinicians.  I strive to shine as a Global Midwife seeking to increase the survival of mothers and babies in sub-Saharan Africa.  Hear me: “I see you mamas and babies, and I care for you; *you are visible*.”  This is my pledge.  Signed……………………………………………. Date………………………………. |
| --- |

Table 0‑2 Midwife Vision Pledge in Swahili (Kiswahili)

| **Ahadi ya Midwife Vision**    Mimi naahidi kushikilia kiwango cha juu cha ujuzi, huduma na mwenendo wa taaluma ya kiliniki.  Mimi wakati wote nitakuwa na huruma nakuzingatia huduma bora kwa mama na watoto watakao kuwa chini ya huduma yangu.  Nakubali kuwa mimi ni sehemu ya timu, na nitasaidia wakunga wenzangu, wauguzi na wataalamu wa kiliniki.  Najitaidi kuangaza kama mkunga wa kimataifa anayetaka kuongeza uokoaji wa maisha ya mama na watoto wachanga Kusini mwa janga la Sahara.  Nisikilizeni: “nakuona wewe mama na mtoto, na ninakujali; *wewe sio usiyeonekana*.”  Hii ni ahadi yangu.  Saini……………………………………………. Tarehe………………………………. |
| --- |
